# Comparative Analysis of Toxigenic and Megaplasmid-Free *Clostridium tetani* Strains Reveals Physiological Transitions Linked to Tetanus Toxin Production

**DOI:** 10.3390/microorganisms14081640

**Published:** 2026-07-27

**Authors:** Bastien Marie, Nathalie Gorret, Raphaël Esson, Romain Pizzato, Dominique Garnier, Stéphane E. Guillouet, Isabelle Meynial-Salles

**Affiliations:** 1 Toulouse Biotechnology Institute, Université de Toulouse, CNRS, INRAE, INSA, 31077 Toulouse Cedex 4, France; b_marie@insa-toulouse.fr (B.M.); ngorret@insa-toulouse.fr (N.G.); guilloue@insa-toulouse.fr (S.E.G.); 2 Sanofi, 69280 Marcy L’Etoile, France; raphael.esson@sanofi.com (R.E.); romain.pizzato2@sanofi.com (R.P.); dominique.garnier@sanofi.com (D.G.)

**Keywords:** *Clostridium tetani*, tetanus toxin, flow cytometry, bacterial morphology, megaplasmid, toxin secretion, anaerobic controlled fermentation

## Abstract

Understanding *Clostridium tetani* metabolism and physiology is essential for identifying factors controlling toxin synthesis. We compared a toxigenic *C. tetani* strain with a derivative strain lacking the toxin-encoding megaplasmid carrying *tetR* and *tetX* genes involved in toxin regulation and production. Both strains were cultured under identical strict anaerobic conditions in complex media. The toxigenic strain exhibited four successive fermentation phases, including rapid growth, toxin accumulation-associated slower growth, cell lysis, and toxin maturation. Cultivation yielded 0.45 g/L of biomass, for a final toxin titer of 45 Lf/mL. The megaplasmid-free strain demonstrated no toxin production or cell lysis, yielding a higher biomass concentration (0.75 g/L) maintained for 150 h. Flow cytometry was applied for the first time to monitor *C. tetani* population dynamics throughout the culture. The toxigenic strain exhibited notable morphological heterogeneity and transient filamentous cells during rapid growth, followed by two smaller-cell subpopulations emerging during toxin production. The megaplasmid-free strain rapidly stabilized into a single homogeneous population. Membrane integrity remained largely preserved, while extracellular toxin became detectable, suggesting that extracellular toxin detection before cell lysis onset may involve an active export mechanism. Overall, these findings reveal the physiological transitions associated with megaplasmid presence and provide new insights into the cellular processes underlying tetanus toxin production.

## 1. Introduction

Tetanus constitutes an acute toxic infection caused by the bacterium *Clostridium tetani* and can affect humans and animals. Owing to its ability to secrete an extremely potent neurotoxin—which is considered one of the most powerful currently identified—this infectious disease remains a major public health problem, particularly in developing countries, with approximately 25,000 deaths from neonatal tetanus being reported in 2018 [1]. To combat the disease, intensive treatment based on antibiotics and immunoglobulin G (IgG) cocktails is adopted [2]. However, the simplest and safest way to protect against tetanus is vaccination, which involves the administration of a detoxified form of the toxin (toxoid) to stimulate the immune system through the use of repeated doses at regular intervals.

*C. tetani* is a strict anaerobic Gram-positive microorganism that possesses a megaplasmid with a size of 74,082 bp, which exhibits a G+C content of 24.5% and 61 open reading frames (ORFs). This plasmid mainly carries genes associated with virulence, notably *tetX* (also referred to *tent*), which encodes for tetanus neurotoxin (TeNT), and *tetR*, which is the associated transcriptional regulator [3]. Because the megaplasmid is large, it is usually present as a single copy per cell. Loss of this plasmid yields a nontoxigenic phenotype [4,5].

Because of its ability to synthesize TeNT, *C. tetani* has been cultivated industrially for decades for vaccine manufacturing. The toxin is subsequently detoxified to produce tetanus toxoid and is also widely employed as a carrier protein for conjugate vaccines. The culture medium used by manufacturers has remained largely unchanged for nearly 70 years. Notably, it is a complex medium that contains peptides derived from casein hydrolysate, beef heart infusion, and glucose [6]. Subsequent improvements eliminated the use of beef heart extract [7]; however, batch-to-batch variability resulting from the poorly defined complex raw materials still leads to variability in manufacturing performance. Without fully eliminating complex components, teams have developed laboratory-scale media based on plant-derived peptones to reduce the occurrence of potential allergic reactions linked to animal-derived compounds [8].

*C. tetani* is cultivated primarily in batch mode, both at the laboratory scale and in industrial production processes. This cultivation mode has facilitated the determination of optimal conditions for biomass and toxin production. Since the 1970s, research has mainly focused on improving culture media and optimizing toxin production, while the overall cultivation strategy has remained largely unchanged. Approximately one decade ago, Licona-Cassani et al. (2016) described three distinct growth phases, followed by a final toxin maturation stage, during the culture of *C. tetani* in complex media [9]. The first phase, which lasted approximately 10 h, corresponded to rapid exponential growth, characterized by increased biomass production and a decrease in pH. Using chemically defined media, the authors also demonstrated that during this period, the bacterium preferentially consumed free amino acids. Partial or complete depletion of these amino acids led to the second phase, characterized by a significant deceleration in growth and the use of peptides, which is more energy-intensive. This phase, lasting approximately 30 h, was associated with progressive alkalization of the medium and activation of *tetX* expression, which is positively regulated by the TetR protein. Finally, a cell lysis phase occurred between 40 and 70 h of culture, followed by a stage in which toxin maturation was observed. While the majority of tetanus toxin is released during the cell lysis phase, no specific export systems have been identified. This contrasts with other toxigenic *Clostridium* species, in which toxin release involves holin-like proteins and endolysins [10,11,12,13,14].

Optimizing TeNT production requires a detailed understanding of *C. tetani* metabolism and physiology. However, important knowledge gaps remain regarding the role of the toxin-encoding megaplasmid and the physiological transitions associated with toxinogenesis and cell lysis. These limitations prevent further optimization of cultivation processes.

To gain further insight, we performed a comparative analysis using two *C. tetani* strains grown under identical conditions: (i) strain A, which is highly similar to the reference strain E88, and (ii) strain G761, which lacks the megaplasmid. To investigate these physiological processes, batch cultures were conducted in reinforced clostridial medium (RCM), a commercially available, ready-to-use complex medium commonly employed for cultivating anaerobic bacteria. Although RCM has never been described for *C. tetani* cultivation, it supports both bacterial growth and tetanus toxin production while providing a simple and reproducible cultivation system. Both the growth kinetics and the exometabolome were characterized. Additionally, for the first time, flow cytometry analysis was applied throughout batch cultures to monitor the physiology of cell subpopulations and cell viability. This comparative approach was designed (i) to investigate whether the presence of the toxin-encoding megaplasmid influences cellular physiology beyond toxin synthesis and (ii) to determine whether toxin production is associated with morphological transitions, membrane integrity, and cell lysis. By integrating metabolic, morphological, and physiological data, this study provides novel insights into the cellular determinants of TeNT production and lays the groundwork for formulating more efficient toxin optimization strategies.

## 2. Materials and Methods

### 2.1. Strains

Two wild-type strains of *C. tetani* were employed, namely (i) *C. tetani* strain A, a derivative of the Harvard strain that is mainly used by European vaccine manufacturers [5,15,16,17], and (ii) *C. tetani* strain G761, which naturally loses the plasmid carrying the TeNT gene cluster [18,19].

### 2.2. Culture Medium

RCM (Merck KGaA, DA) was used for liquid culture purposes. It contains meat extract (10 g/L), peptone (10 g/L), yeast extract (3 g/L), D(+)-glucose (5 g/L), starch (1 g/L), sodium chloride (5 g/L), sodium acetate (3 g/L), and L-cysteine hydrochloride (0.5 g/L). Additional D(+)-glucose was added to reach a final concentration of 18 g/L. The pH was adjusted to 7.2 using a 1 M NaOH solution. Gaseous nitrogen flow was gently bubbled into the liquid medium for 15 min to reduce the concentration of dissolved oxygen. The medium was then autoclaved for 15 min at 121 °C, after which it was fully degassed.

### 2.3. Cultivation Conditions

#### 2.3.1. Inoculum Standardization

To standardize each culture, a propagation chain was implemented prior to each experiment in flasks or bioreactors. A single glycerol stock stored at −80 °C was thawed and used to inoculate a screw-cap tube containing 9 mL of degassed RCM. The tube was incubated in a water bath (IsoTemp GPD10, Fisherbrand^TM^, Thermo Fisher Scientific, Waltham, MA, USA) at 34.5 °C for 24 h until reaching optical density OD_600nm_ values varying between 0.5 and 1.0 U (refer to Section 2.4). After gentle homogenization, the cell suspension was withdrawn and used for deep inoculation in a 100 mL Schott^®^ flask containing 80 mL of RCM (2% *v*:*v*). This flask was incubated at 34.5 °C, without shaking, for 24 h, until the same OD_600nm_ range was attained (0.5–1.0 U). Deep inoculation limits oxygen transfer, which could otherwise disrupt strain growth.

#### 2.3.2. Flask Cultures

The final propagation step (refer Section 2.3.1) was implemented to inoculate a 250 mL Schott^®^ flask containing 200 mL of RCM (3.5% *v*:*v*). The cultures were incubated at 34.5 ± 1 °C under magnetic stirring at 100 rpm in a dry bath (2 mag AG Magnetic Motion, München, Germany). A surface nitrogen flow was applied to maintain anaerobic conditions, and the flow rate was independently set to 500 mL/h for each flask using rotameters (Sho-Rate^TM^, Brooks Instrument^®^, Hatfield, PA, USA). The flasks were fitted with four-port caps: two ports were for gas inlet and exhaust purposes, one equipped with a Luer-lock was used for inoculation or additions, and one was connected to a vacuum sampling system (Vacutest^®^ Kima, Arzergrande, Italy).

#### 2.3.3. Bioreactor Cultures—Batch Mode

Bioreactor cultures were performed using a Sartorius Biostat B-DCU system (Sartorius AG, Göttingen, Germany) equipped with a 3 L glass vessel (240 mm height × 130 mm diameter) containing 1.5 L of RCM. The medium was inoculated with the final propagation culture (refer to Section 2.3.2; OD_600nm_ range = 0.5–1.0 U) at 3% (*v*:*v*). The pH was monitored using a gel-filled pressurized probe (405.DPAS-SC K8S, Mettler Toledo, Columbus, OH, USA) and maintained at 6.8 ± 0.05 through the automated addition of 1 M KOH or 1 M HCL. The temperature was maintained at 34.5 °C using a probe (Biostat B-DCU system) facilitating monitoring and control through a water jacket connected to a cryostat (9110-BB, PolyScience, Niles, IL, USA) set to 8 °C. Both pH and temperature were controlled using a proportional-integral-derivative (PID) controller (Biostat B-DCU system). A RedOx probe (InPro 3100I/SG, Mettler Toledo, OH, USA) was used for redox monitoring. Its signal was adopted independently using an external transmitter (M400/T2, Mettler Toledo, OH, USA). Stirring was conducted at 100 rpm using a marine impeller. Anaerobic conditions were ensured with a surface nitrogen flow of 750 mL/min. Data were acquired using MFCS/win 2.1 software (Sartorius AG, Göttingen, Germany), except for the redox potential measurements.

### 2.4. Biomass Characterization

The cell density was estimated by measuring turbidity at 600 nm using a spectrophotometer (Libra S4 Visible, Biochrom, Cambridge, UK) and 1 cm path-length semimicro polystyrene cuvettes (BioSigma, Cona, Italy). Osmosis water was employed as the blank. The samples were diluted with osmosis water such that their absorbances remained within the linear range of the instrument (0 to 0.6 absorbance units). A previously established, laboratory correlation was adopted to convert the OD_600nm_ value to the cell dry weight (1 U_OD600_ = 0.321 g_X_/L; unpublished data). For this purpose, Eppendorf tubes were dried in a vacuum drying oven (60 °C, 200 mbar; Vacutherm, Warren, VT, USA) and weighed on a precision balance (±0.01 mg, XP6 Mettler Toledo, OH, USA). Three milliliters of culture broth were transferred into preweight tubes and centrifuged (3 min, 8000 rpm). The supernatants were discarded, and pellets were washed twice using Milli-Q water before being dried again in a vacuum drying oven. The resulting mass differences were then plotted against OD_600nm_ to establish a linear correlation.

### 2.5. Microscopy

Fresh culture broth samples were fixed on microscope slides. Gram staining (VWR, Radnor, PA, USA) was conducted before observing the samples under a light microscope (B–510PH, Optika, Ponteranica, Italy) equipped with a camera (Optikam PRO6, Optika, Ponteranica, Italy).

### 2.6. Flow Cytometry

Single-cell analyses were performed using a BD Accuri C6^®^ flow cytometer (BD Biosciences, Franklin Lakes, NJ, USA) equipped with blue (488 nm) and red (640 nm) excitation lasers. Forward scatter (FSC: 0 ± 13°) and side scatter (SSC: 90 ± 13°) signals were collected and employed as proxies for the cell size and granulometry, respectively [20]. Fluorescence was measured using four photomultiplier detectors, namely FL1 (533/30 nm), FL2 (585/40 nm), FL3 (>670 nm), and FL4 (675/25 nm).

Cell viability was assessed using the LIVE/DEAD BacLight^TM^ Bacterial Viability Kit (Invitrogen, Waltham, MA, USA). SYTO 9^TM^ staining provides bacterial nucleic acids with green fluorescence, whereas propidium iodide penetrates only cells with compromised membranes, and such cells emit red fluorescence. As all *C. tetani* cells were SYTO 9-positive, noncellular events could be excluded. SYTOX^TM^ Deep Red was additionally employed as an alternative membrane-impermeant dye.

Data were acquired using BD Accuri CFlow^®^ software version 1.0.264.14. Samples were analyzed up to a count of 20,000 events at a low flow rate (14 µL/min) using Milli-Q water as the sheath fluid. FSC and SSC signals were used as trigger channels, with 12,000 and 2000 as thresholds, respectively. Data processing was performed using FlowJo (Becton Dickinson, Ashland, NJ, USA). The singlet method was applied to exclude doublets, aggregates, or debris. Singlets were identified using FSC-A/FSC-H and SSC-A/SSC-H plots. Singlets exhibited a linear correlation between A and H, whereas doublets appeared as events with disproportionate values [20].

### 2.7. Metabolite Quantification

#### 2.7.1. Determination of Organic Acid, Solvent and Glucose Concentrations

The concentrations of glucose, organic acids (acetate, butyrate, pyruvate, succinate, citrate, lactate, propionate), and solvents (ethanol, butanol, acetone) were determined from culture supernatants using a high-performance liquid chromatography (HPLC) instrument (1260 Infinity II, Agilent, Santa Clara, CA, USA) equipped with a 1260 quaternary pump VL G7111A, a vial sampler G7129A, an integrated column compartment G7130A, a variable wavelength detector G7114A, and a refractive index detector G7162A. Separation was achieved using a system comprising a micro-guard cation H refill cartridge, 30 × 4.6 mm (Bio-Rad, Hercules, CA, USA), and an Aminex HPX–87H column, 300 × 7.8 mm, with a 9 µm particle size (Bio-Rad, CA, USA). Chromatographic data were acquired, analyzed, and quantified in OpenLab software (version 2.7, Agilent, CA, USA). Quantification was based on calibration curves prepared from analytical standards. The masses of the standard components were measured with an accuracy of 10^−3^.

#### 2.7.2. Fermentation Gases

Fermentation gases were measured online by connecting the gas outlet to a micro gas chromatograph. The experimental system encompassed a 990 Micro GC instrument (Agilent, CA, USA) equipped with two separating columns and a thermal conductivity detector (TCD). The Cox UM column (1 m × 0.8 mm, injector temperature 80 °C, column temperature 79 °C; Agilent, CA, USA) facilitated the separation of hydrogen (H_2_) and carbon dioxide (CO_2_). The second column, a PoraPlot U FS (10 m × 0.25 mm × 8 µm, injector 70 °C, column 100 °C; Agilent, CA, USA), enabled the separation of hydrogen sulfide (H_2_S), methanethiol (CH_3_SH), and carbon dioxide (CO_2_). In both columns, helium was used as the gas vector at a pressure of 200 kPa. Quantification was performed using a calibration gas mixture comprising 0.3% H_2_, 1.5% CO_2_, 500 ppm H_2_S, and 500 ppm CH_3_SH in nitrogen (Airproducts, Allentown, PA, USA).

#### 2.7.3. Toxin Concentration Determination

##### Ramon Flocculation

Toxin titration was performed using the Ramon flocculation assay applied to freshly filtered supernatants, as previously described for diphtheria toxin [21]. Toxin titers are expressed as the limit of flocculation per mL (Lf/mL).

##### ELISA

A sandwich enzyme-linked immunosorbent assay (ELISA) was employed to quantify the native toxin on the basis of the of the antigenic epitopes shared between the toxin and toxoid forms. Nunc MaxiSorp c96 plates (Thermo Scientific, MA, USA) were coated with 100 µL of polyclonal anti-TetX antibody (goat, Fisher Scientific, Waltham, MA, USA) at a concentration of 10 µg/mL in phosphate-buffered saline (PBS), followed by overnight incubation (~15 h) at 4 °C. The plates were then washed three times with 300 µL of wash buffer (PBS + 0.05% Tween20). The wells were blocked with 200 µL of blocking buffer (PBS containing 1% skim milk) and incubated for 1 h at 37 °C. After the cells were washed, 100 µL of blocking buffer was added to each well. A standard or samples (100 µL) were added to the first well of each row. After homogenization, twofold serial dilutions were implemented across the plate by transferring 100 µL from one well to the next, up to column 11, corresponding to a 2048-fold dilution. The final column, which contained blocking buffer only, served as a blank. The plates were incubated for 1 h at 37 °C.

Following an additional washing step, 100 µL per well of monoclonal anti-TetX antibody (18E11 from mice, Envigo, Indianapolis, IN, USA) at a concentration of 10 µg/mL in blocking buffer was added and incubated for 1 h at 37 °C. The plates were subsequently washed again and incubated with 100 µL of 0.08 µg/mL polyclonal anti-mouse horseradish peroxidase (HRP)-conjugate antibody (from goat, Jackson ImmunoResearch, West Grove, PA, USA) before incubating for 30 min at 37 °C. After the final washing step, 100 µL of 3,3′,5,5′-tetramethylbenzidine TMB substrate (Bio-Rad, CA, USA) was added to each well. The enzymatic reaction occurred at room temperature in the dark and was stopped after 30 min through the addition of 100 µL of 1 M HCl, resulting in a color change from blue to yellow. The absorbance was measured at 450 nm. The OD value is proportional to the concentration of toxin or toxoid detected by the antibody pair.

Toxin or toxoid concentrations were back-calculated using a reference standard-dose–response curve calibrated in units of Lf/mL. The reference standard comprised samples from a batch culture grown in Mueller and Miller medium, titrated at 40 Lf/mL using the Ramon flocculation method, and verified every 6 months. Purified anatoxin (4250 Lf/mL) was included on each plate as an internal control.

The calibration curve was generated by plotting the absorbance as a function of the toxin concentration on a logarithmic *x*-axis and fitted based on the basis of a typical dose–response equation, which can be defined as follows:

*y* = D + (A − D)/(1 + (*x*/C))^B^

Therefore, the following can be obtained:

*x* = C × ((A − D)/(*y* − D) − 1)^(1/B)^

where A denotes the lower asymptote, B denotes the slope, C denotes EC50, and D denotes the upper asymptote.

Toxin quantification was performed for both extracellular and intracellular fractions derived from identical culture samples. Following centrifugation, the supernatants were employed directly for extracellular toxin determination, and the results are expressed as Lf/mL of culture supernatant. The corresponding cell pellets were resuspended and disrupted through sonication (FB120, Fisherbrand^TM^, Waltham, MA, USA) to release intracellular toxin, which was subsequently quantified. Intracellular toxin concentrations are also expressed in units of Lf/mL, corresponding to the theoretical concentration that would have been measured if the toxin had been released into the culture medium.

### 2.8. Data Treatment

The state variable measurements are presented with their 95% confidence intervals, which were determined beforehand on the basis of five technical replicates. The error of the specific growth rate (determined as being the slope of ln[Optical Density] = f(t)) was calculated as the standard deviation (SD) of the slope. The instantaneous specific growth rates were calculated upon fitting of the experimental data and the derivative calculations. For the yield determination, the state variables were plotted pairwise in a scatter plot within the considered period of the culture. A linear regression was applied to determine the yield (as the slope) and the error (as the SD of the slope). Specific growth rates (h^−1^), substrate and product yields (Y_S_, g_X_/g_S_ and Y_P_, g_P_/g_X_, respectively), and production/consumption rates (g/L/h) were calculated for each growth phase. The rates observed during culture were expressed on a carbon-molar basis, by relating them to the number of moles of carbon contained in each compound (expressed in units of Cmol/L/h). Biomass was converted from g_X_ to Cmol_X_ using the following elemental composition, previously determined in our laboratory (unpublished data): CH_1.780_O_0.490_N_0.250_S_0.010_. With respect to the toxin, a correlation of 1 Lf/mL = 2 µg/mL was applied. Conversion from g_Tox_ to Cmol_Tox_ was performed using the following elemental formula for tetanospasmin: CH_1.841_O_0.477_N_0.280_S_0.028_. The latter was calculated empirically on the basis of the average content of proteinogenic amino acid and the molecular mass of tetanospasmin (150 kDa), which was derived from its amino acid composition. Notably, the biomass production is very low under anaerobic fermentation conditions. Compared with fermentative end-products, the concentrations of toxin are negligible. Consequently, the approximations made for the toxin elemental composition minimally influence the carbon and elemental balance results.

The carbon balance was therefore established as follows:

∑(r_i_^C^ = 0)

where r is the rate associated with compound i involved in the biological reactions. Notably, r is expressed in units of Cmol_i_/L/h.

The elemental balance is based on the same principle applied to all compounds containing at least one of the elements C, H, O, and N and can be described as follows:

∑(*γ*_i_ × r_i_^CHON^ = 0)

where *γ* is the generalized degree of reduction of compound i [22]. Here, r is expressed in units of Cmol_i_/L/h for carbon-containing compounds or in units of mol_i_/L/h for compounds lacking carbon.

## 3. Results and Discussion

To determine the distinct characteristics of toxigenic and megaplasmid-free *C. tetani* strains, we first aimed to determine their growth features via flask culture experiments.

### 3.1. Growth Kinetics

Preliminary flask cultures were first conducted (as described in Section 2.3.2) to assess whether RCM could support the growth of both strains and facilitate tetanus toxin synthesis by strain A. Under flask conditions, *C. tetani* strain A reached an OD_600nm_ value of 2.8 after 50 h of cultivation. The growth process proceeded through two distinct phases, with an initial exponential growth rate of µ = 0.23 ± 0.003 h^−1^, followed by a slower growth phase (µ = 0.016 ± 0.002 h^−1^). Tetanus toxin was detected through ELISA, confirming that RCM supports toxin synthesis. However, the toxin concentration could not be accurately quantified, as only low levels of extracellular toxin were measured. Cell lysis was not observed for strain A within the cultivation window. The biomass of strain G761 increased, reaching an OD_600nm_ value of 4.1 after 50 h, with growth rates of 0.20 ± 0.003 h^−1^ in Phase I and 0.036 ± 0.0004 h^−1^ in Phase II.

RCM was subsequently selected for batch cultivation in a bioreactor, as it provides an easily implementable and commercially available complex medium suitable for clostridial growth, despite not having been previously described for *C. tetani*. While both RCM and the historical Mueller and Miller medium supply complex nitrogen sources through meat-derived extracts and peptones, they differ substantially in terms of composition and intended purpose. The Mueller and Miller formulation was specifically designed and optimized for *C. tetani* growth and high toxin yields, whereas RCM contains lower concentrations of mineral salts and lacks several components of the ancestral medium, including the vitamin mixture and uracil [6]. In addition, RCM contains sodium acetate but does not contain L-tyrosine. Consequently, RCM represents a simpler and less enriched medium that supports growth and toxin synthesis but is not expected to reproduce the physiological optimization achieved with the ancestral formulation.

**Figure 1:**
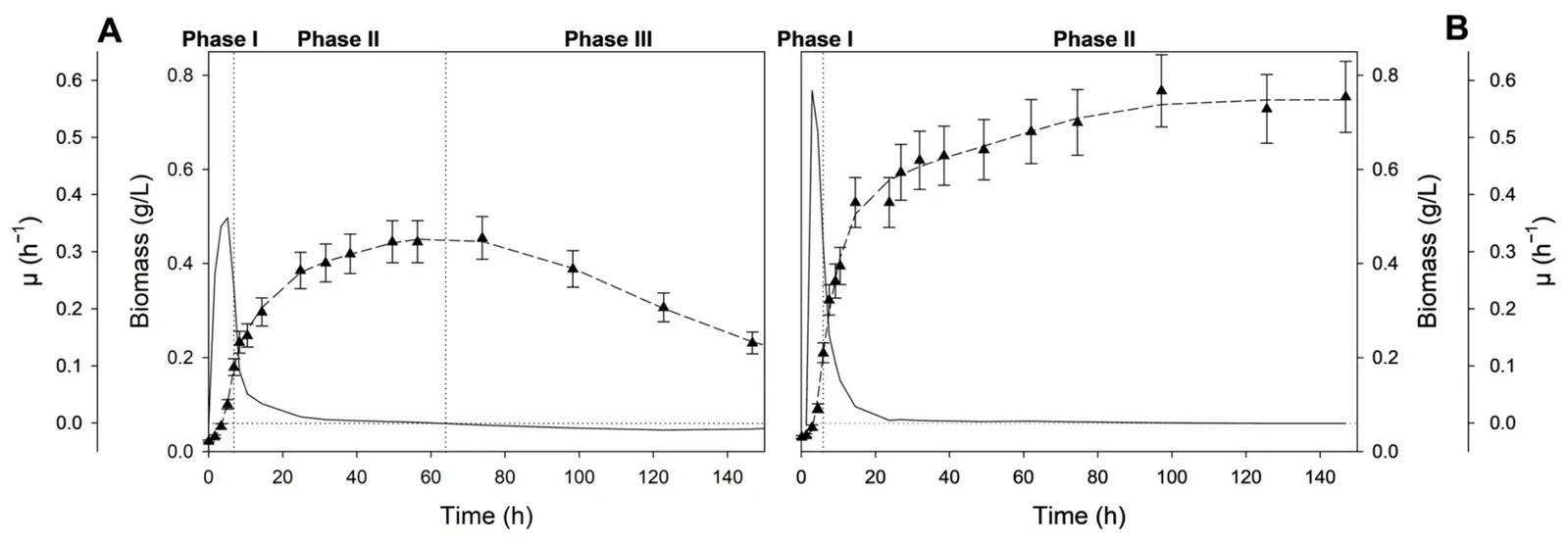
Biomass concentration and production rates of *C. tetani* strain A (**A**) and *C. tetani* strain G761 (**B**) in RCM under batch mode culture conditions in a bioreactor. (▲) Biomass; (−) growth rate, µ.

**Figure 2:**
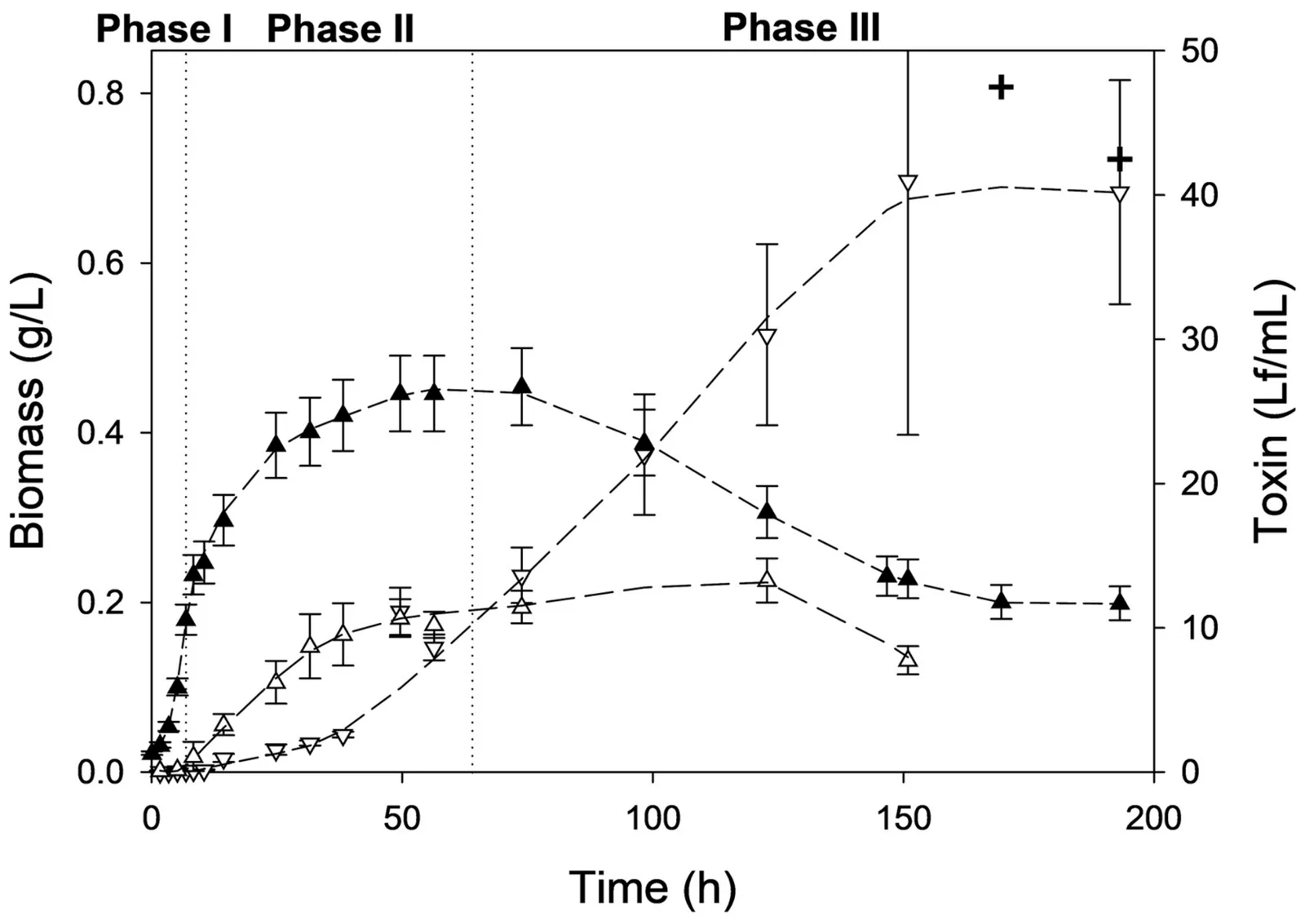
Biomass concentrations and toxin levels from *C. tetani* strain A in RCM under batch mode culture conditions in bioreactor. (▲) Biomass; (△) Intracellular toxin (ELISA); (▽) extracellular toxin (ELISA); (+) extracellular toxin (Ramon method).

*C. tetani* strain A cultivated in RCM exhibited growth kinetics comparable to those previously reported by Licona-Cassani et al. (2016) [9]. Three distinct phases were observed, namely (i) an initial exponential growth phase (Phase I), (ii) a slower growth phase during which toxin synthesis and accumulation occurred (Phase II), and (iii) a cell lysis phase associated with toxin release (Phase III) (Figure 1A). During Phase I, which lasted approximately 7 h, exponential growth occurred at an average growth rate of 0.34 ± 0.0007 h^−1^, leading to 0.18 g/L of biomass formation. Medium acidification was achieved through the addition of NaOH, which was automatically supplied to maintain the pH at 6.8. After 7 h of culture, a reversal in the pH trend was observed, indicating that metabolic shifts occurred in the culture and that pH regulation subsequently necessitated the addition of HCl. During Phase II, the growth rate decreased to 0.051 ± 0.001 h^−1^. However, the growth process continued to decelerate progressively, reaching µ = 0.005 ± 0.0002 h^−1^. This further decrease suggests that factors beyond the metabolic burden of toxin production may restrict growth at this stage. Possible explanations include nutrient limitations, the accumulation of inhibitory metabolites, and suboptimal metabolic adaptation to the culture conditions. A maximum biomass value of 0.45 g/L was obtained after 75 h of fermentation, prior to the onset of Phase III. At the lysis stage, the OD decreased progressively, and tetanus toxin was released into the extracellular environment. The biomass concentration subsequently stabilized (Figure 2), indicating a phase involving extracellular toxin maturation [9].

*C. tetani* strain G761, which lacks the megaplasmid, exhibited a similar growth profile at the early culture stages (Figure 1B). Phase I was characterized by a relative high average growth rate (µ = 0.42 ± 0.014 h^−1^), resulting in 0.21 g/L of biomass at the end of this stage. The maximum biomass production rate, r_Xmax_, reached 0.065 g_X_/L/h, which is approximately 55% greater than that of the toxigenic strain. Thereafter, the decrease in the growth rate observed in both strains indicated a metabolic shift associated with the transition to Phase II, as also supported by the observed pH inversion. The growth rate decreased to 0.071 ± 0.005 h^−1^ within approximately 7 h before decreasing further to 0.004 ± 4 × 10^–5^ h^−1^ over the subsequent 60 h.

Overall, the biomass kinetics were consistently greater in the plasmid-free strain. Although limited information on megaplasmids is available, plasmids are generally recognized to impose an energy burden on host cells, potentially reducing growth rates [23]. In the absence of the megaplasmid, strain G761 likely experiences reduced metabolic costs associated with plasmid maintenance and gene expression, which may partly explain the higher growth rates observed. However, additional regulatory effects associated with plasmid loss cannot be excluded. The absence of plasmid-encoded regulatory functions may indirectly influence chromosomal gene expression and metabolic regulation, thereby contributing to the observed physiological differences.

In contrast to strain A, strain G761 did not experience cell lysis after 150 h of culture. Gram staining confirmed the absence of cell debris, which indicates that cellular integrity was maintained throughout the process. The biomass concentration stabilized at 0.75 g/L after 100 h of culture, highlighting a key difference between the two strains. Notably, whereas strain A experienced cell lysis as part of its toxin-associated life cycle, strain G761 remained stable and achieved a higher final biomass concentration.

### 3.2. Toxin and Cell Lysis

To characterize the relationship between growth, cell lysis, and toxin production, tetanus toxin concentrations were monitored throughout cultivation. The final tetanus toxin levels in the *C. tetani* strain A cultures were quantified and reached approximately 45 Lf/mL between 170 and 193 h of cultivation using the Ramon flocculation method. This method, historically employed for tetanus toxin quantification, provides reliable measurements at moderate to high concentrations but exhibits limited accuracy at low concentrations, performing optimally within the 30–100 Lf/mL range [24].

To monitor toxin synthesis throughout the culture process and to accurately quantify both the intracellular and extracellular toxin fractions, an ELISA was employed in parallel. ELISA, which requires smaller sample volumes, offers higher sensitivity for measuring low toxin concentrations. This method is officially recognized by the World Health Organization (WHO) for quantifying the content of antigens and degree of adsorption in the final vaccine products, with a reported detection limit of 0.002 Lf/mL [25]. Intracellular toxin accumulation began at the onset of Phase II and progressively increased during this period, reaching approximately 11 Lf/mL prior to entry into Phase III. The depletion of specific amino acids during Phase I or the consumption of peptides during Phase II may induce toxin synthesis, potentially through TetR overproduction [9]. Toxin release into the extracellular environment was first observed at approximately 15 h of culture, although the concentration remained below 3 Lf/mL until 45 h. A notable increase then occurred concomitant with growth deceleration, leading to complete toxin release by the end of Phase III (Figure 2). Though ELISA has limited precision for native toxin quantification, its high sensitivity enabled toxin dynamics to be monitored throughout the culture process. Combined with the separate analysis of intracellular and extracellular samples, this approach allowed discrimination between both toxin fractions.

Although RCM supported toxin synthesis, it was not optimal for achieving high toxin concentrations (70 to 120 Lf/mL), in contrast to modified Massachusetts medium, which does not contain meat extract [7,26,27]. The growth deceleration observed during Phase II may reflect nutritional limitations, which could also contribute to premature extracellular toxin release.

**Figure 3:**
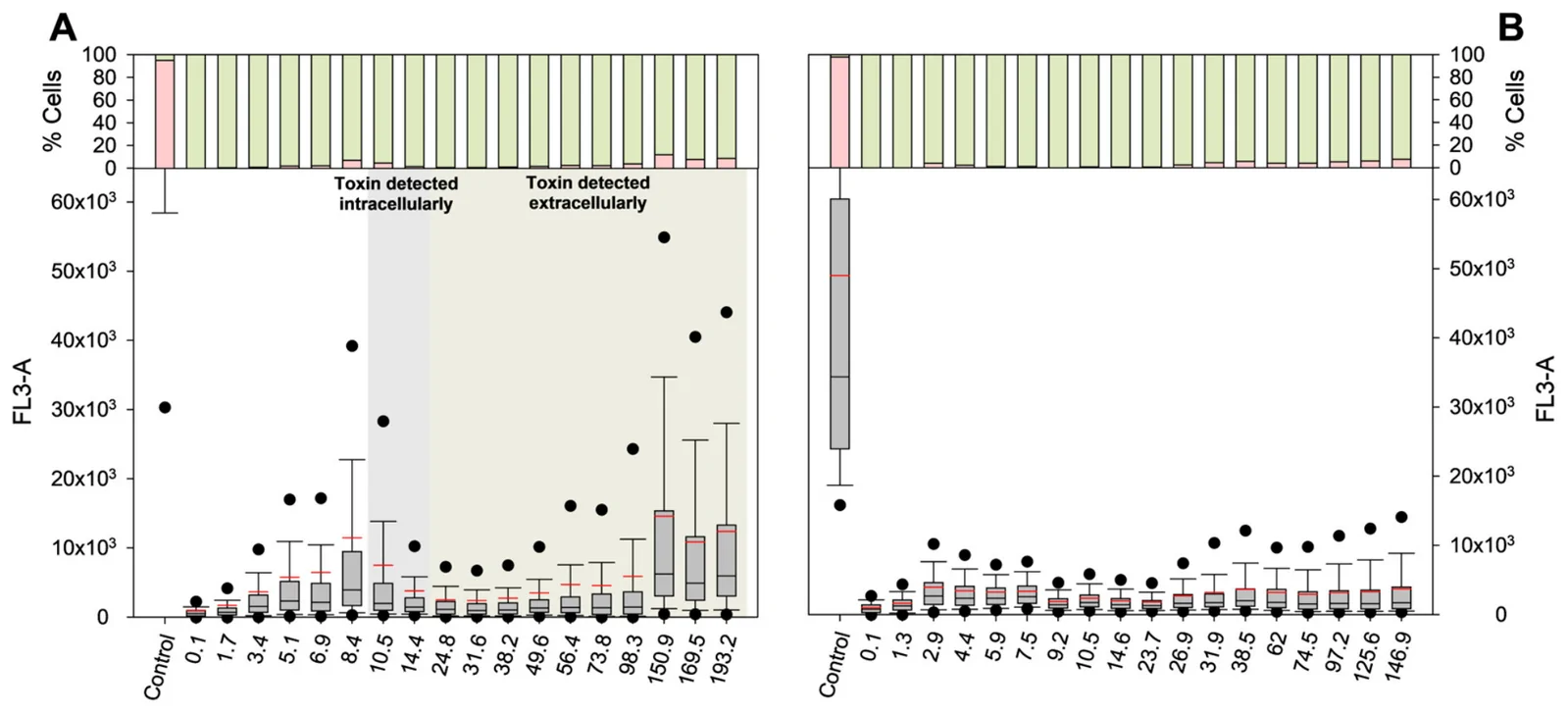
Percentage of nonintact cells and distribution of the FL3-A signal (propidium iodide fluorescence) over time in *C. tetani* strain A (**A**) and *C. tetani* strain G761 (**B**) during batch culture in RCM. A positive control was obtained after the cells were exposed to heat shock (90 °C for 1.5 h). However, its complete signal is not fully shown to avoid overwriting the kinetic data. (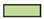) Viable cell fraction; (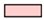) permeabilized cell fraction. This figure shows a classical boxplot representation; the boundary of the box closest to zero indicates the 25th percentile, a black line within the box marks the median, a red line within the box marks the mean, and the boundary of the box farthest from zero indicates the 75th percentile. Whiskers above and below the box indicate the 90th and 10th percentiles. The black dot indicates the 95th and 5th percentiles.

Because toxin release occurred primarily during Phase III, flow cytometry analysis was employed to assess membrane integrity and determine whether toxin diffusion could result from membrane damage prior to cell lysis (Figure 3). Propidium iodine fluorescence, which is indicative of membrane permeability, was monitored over time on the basis of the FL3-A signal. The positive control was generated by heating *C. tetani* cells to 90 °C for 1.5–3 h, resulting in complete membrane permeabilization and high PI fluorescence (~10^5^ FL3-A; Figure 3). During the culture of *C. tetani* strain A, the percentage of permeable cells remained low, increasing from 1.7% to 12% between 50 and 150 h. However, because a threshold of 12,000/2000 (FSC-H/SSC-H) was applied, cell debris was excluded, thereby likely underestimating overall cell mortality. An analysis of the lower regions of Figure 3A,B, which represent the fluorescence distribution in the FL3-A channel, revealed that strain A exhibited a more heterogeneous population, particularly at the end of Phase I and during the cell lysis phase. Combined with observations of biomass reduction, these data suggest that cell lysis is associated with moderate membrane permeabilization and increased FL3-A signal heterogeneity. Comparable results were obtained when SYTOX-Red was applied as an alternative membrane permeability marker (FL4-A, data available upon request).

Toxin was detected intracellularly after 8.5 h of culture and became detectable in culture medium after 15 h, albeit at low concentrations. During most of Phase II, very few permeable cells (<1%) and minimal population heterogeneity were observed. These observations suggest that the presence of toxin in the extracellular medium is unlikely to be linked to generalized membrane alteration. Several explanations are therefore possible, notably the involvement of unconventional toxin export mechanisms. However, low-level cell lysis, falling below the flow cytometry detection threshold, or localized cell damage cannot be ruled out. During Phase III, toxin release likely occurred primarily through cell lysis.

Although no specific secretion system has yet been identified in *C. tetani*, tetanus toxin lacks an N-terminal signal peptide targeting canonical bacterial secretion pathways, suggesting that nonclassical secretion mechanisms may be involved [28]. Unconventional toxin export systems have been described in other toxigenic *Clostridium* species. In bacteria producing large clostridial glucosylating toxins (LCGTs), phage-like holin-endolysin systems mediate toxin release. Holin-like proteins encoded within pathogenicity loci, such as TcdE in *Clostridioides difficile*, TpeE in *Clostridium perfringens*, and TcsE in *Paeniclostridium sordellii*, are essential for toxin secretion [10,12,13]. More recently, a lysin-like protein encoded on the *C. difficile* chromosome was reported to facilitate toxin secretion without inducing cell lysis [14].

Strains lacking the megaplasmid, and therefore the operon needed for toxin synthesis, are nontoxigenic [3]. Consistent with previous reports, megaplasmid loss in *C. tetani* results in a nontoxigenic phenotype [4,5]. As expected, no toxin was identified during the cultivation of *C. tetani* strain G761. Only a small proportion of permeable cells was observed, increasing to approximately 7.5% during the stationary phase at 147 h of culture. Moreover, the population remained homogeneous in terms of FL3-A fluorescence. When correlated with biomass kinetics and light microscopy observations, this membrane permeability was associated with growth arrest rather than with cell lysis.

The absence of cell lysis in strain G761, a not previously reported phenomenon, suggests that lysis in toxigenic strains may be linked to multiple factors, including (i) toxin accumulation above a critical threshold, potentially identified through quorum-sensing or alternative signaling pathways, and/or (ii) the expression of specific genes, such as autolysins or regulatory cascades that directly or indirectly control lytic processes. To date, no environmental trigger has been identified, supporting the hypothesis that cell lysis is primarily physiologically regulated [9].

### 3.3. Glucose

Glucose is the only carbohydrate metabolized by *C. tetani*, primarily through glycolysis, leading to the formation of pyruvate, ATP, and NADH [29]. Under anaerobic conditions, NAD^+^ is regenerated through the fermentative conversion of pyruvate into end products such as acetate, butyrate, and alcohols. The bacterium also possesses genes encoding the pentose phosphate pathway on its chromosome [29]. Nevertheless, this pathway likely supports biosynthetic functions rather than serving as a major route for energy generation.

**Figure 4:**
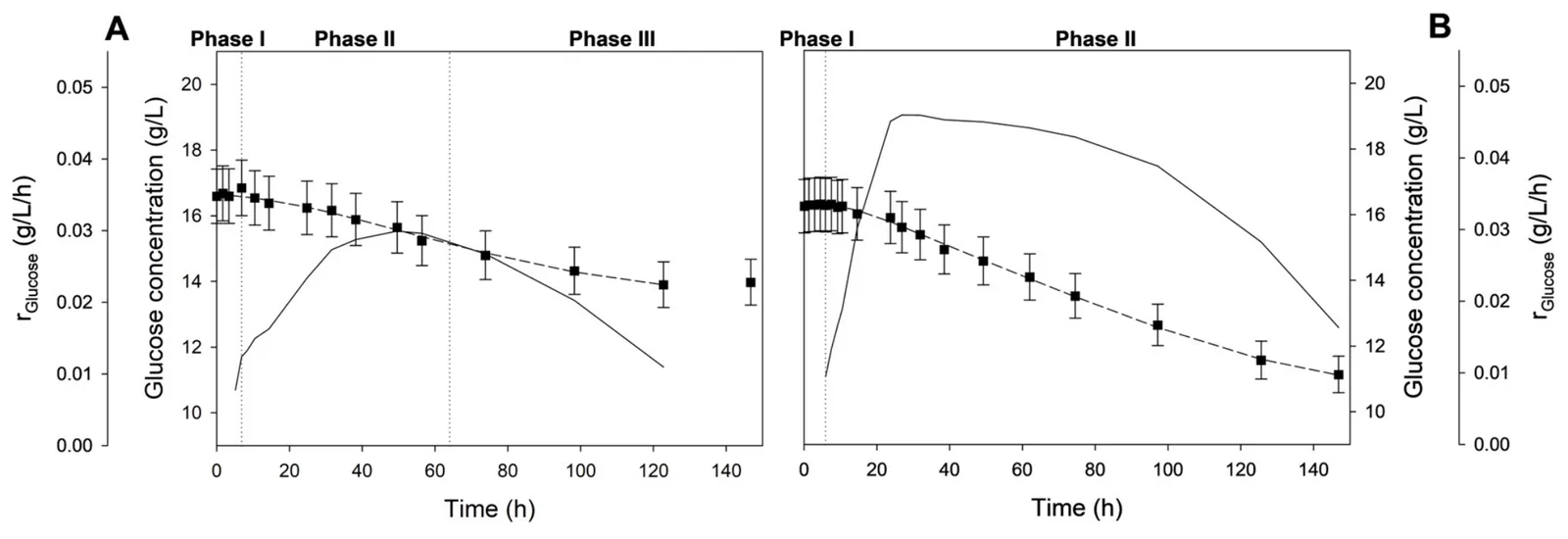
Glucose concentration and consumption rates from *C. tetani* strain A (**A**) and *C. tetani* strain G761 (**B**) in RCM under batch mode culture conditions in a bioreactor. (■) Glucose; (−) glucose consumption rate, r_Glu_.

The Initial glucose concentration was set to 18 g/L, a level far in excess, to ensure that glucose availability did not limit bacterial growth or metabolism. In this study, only glucose was quantified as a carbon substrate, as peptide compounds require preliminary hydrolysis, which was not implemented. Despite this limitation, glucose consumption provides a relevant proxy for primary carbon uptake and facilitates a comparison of cultures. For both strains, glucose consumption began during Phase II, followed by exponential growth, and contributed to overall biomass accumulation. This behavior was also reported by Martinez and Rittenberg (1959) [30]. A level of approximately 2.7 g/L glucose was metabolized by *C. tetani* strain A, with consumption ceasing at the onset of cell lysis (Figure 4A). In contrast, glucose consumption in *C. tetani* strain G761, which did not experience lysis, continued throughout Phase II, reaching a total of 5.2 g/L (Figure 4B). Toward the end of cultivation, the glucose consumption rate (r_Glucose_) decreased markedly, suggesting that residual glucose was used primarily for cell maintenance rather than for biomass production.

### 3.4. Gases

CO_2_ and H_2_ constitute major fermentation products resulting from pyruvate decarboxylation and electron transfer reactions during anaerobic metabolism. Although *C. tetani* can convert glucose into pyruvate through glycolysis, it clearly prefers peptide substrates [29]. In addition, H_2_S and CH_3_SH are produced from the metabolism of cysteine and methionine, respectively [27].

**Figure 5:**
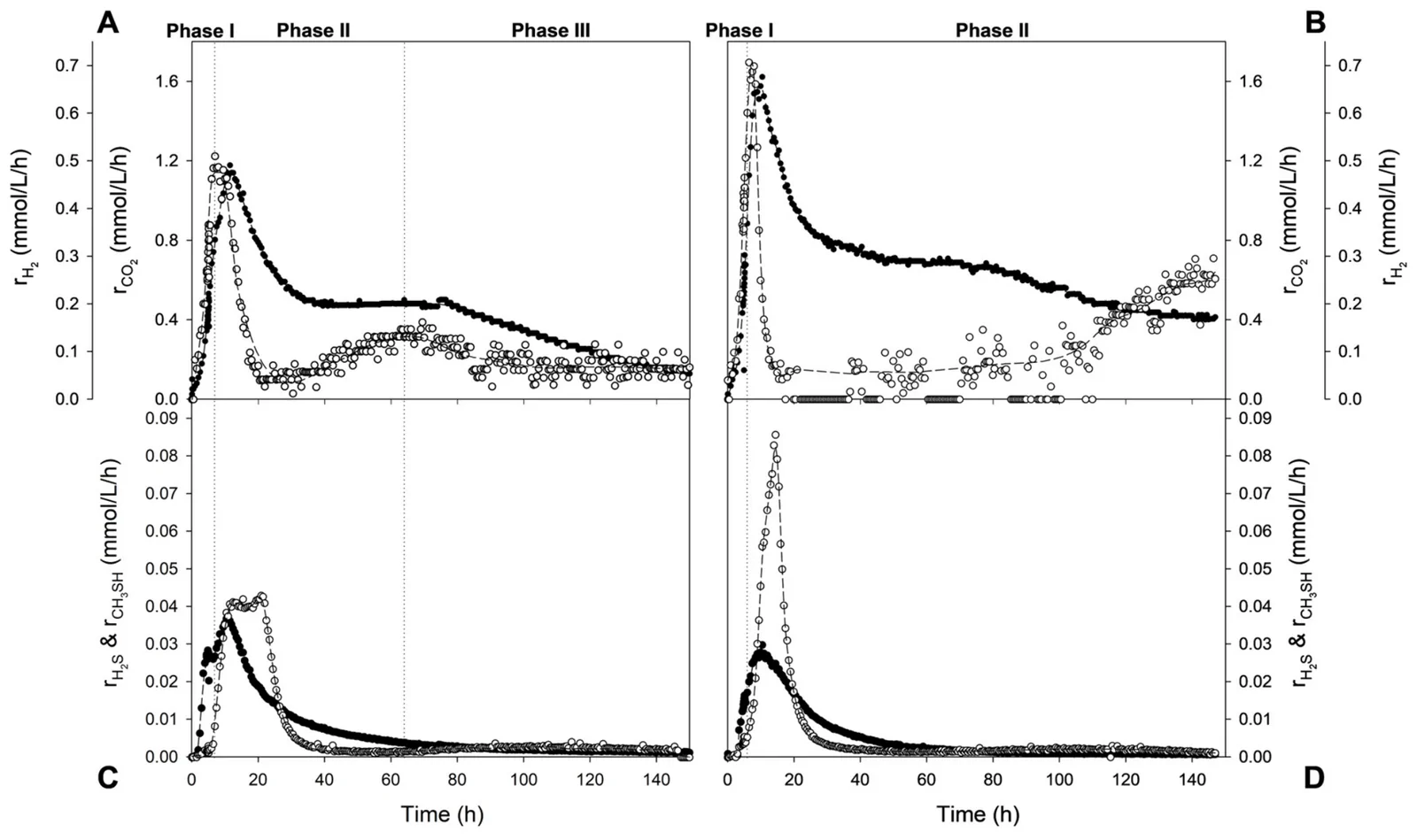
Gas production rates from *C. tetani* strain A (**A**,**C**) and *C. tetani* strain G761 (**B**,**D**) in RCM under discontinuous batch mode culture conditions in a bioreactor. (●) A and B: CO_2_; C and D: H_2_S. (○) A and B: H_2_; C and D: CH_3_SH.

During the initial hours of cultivation, the metabolic behaviors of both strains were similar across all the measured gas production rates (Figure 5). The first exponential biomass growth phase was associated with rapid increases in CO_2_, H_2_, and H_2_S production levels. As expected, both r_CO2_ and r_H2_ were higher in the plasmid-free strain, which reflects its higher growth rate. In contrast, r_H2S_ was lower (maximum value of 0.028 mmol_H2S_/L/h) than in *C. tetani* strain A (0.036 mmol_H2S_/L/h), suggesting a reduced cysteine requirement in the plasmid-free strain.

During Phase II, the metabolic profiles of the two strains differed notably. Only toxigenic strain A exhibited a considerable increase in H_2_ production, with r_H2_ increasing from its post-exponential minimum to 0.150 mmol _H2_/L/h, whereas r_CO2_ remained stable (Figure 5A). This dissociation between r_H2_ and r_CO2_ suggests a redistribution of intracellular electron flow. One possible explanation is the activation of metabolic pathways that generate excess reducing equivalents without proportional decarboxylation. In *Clostridia*, peptide catabolism facilitates the channeling of carbon through pyruvate:ferredoxin oxidoreductase (Pfor) and the butyrate pathway, both of which generate reduced ferredoxin (Fd_Red) as a central redox intermediate. In *Clostridium acetobutylicum*, Pfor and hydrogenases function as tightly coupled modules, with hydrogenases functioning as an electron sink to reoxidize Fd_Red [31]. A similar mechanism likely operates in *C. tetani* strain A: the increased level of H_2_ release suggests a higher intracellular burden of reduced ferredoxin, which must be dissipated via hydrogenases and/or the RNF complex, thereby regenerating NAD^+^ cofactors.

This redox reorientation coincides with the onset of TeNT synthesis. Licona-Cassani et al. (2016) demonstrated that toxin production begins when an organism transitions from the use of free amino acids to peptide utilization, a metabolic switch that notably alters the intracellular electron flux [9]. The present observations extend this model, suggesting that the induction of the TeNT operon is associated with a broader reconfiguration of redox metabolism, which is manifested as increased hydrogenase activity and H_2_ export levels. Consistent with this interpretation, Gregg et al. (2024) proposed that the toxin gene cluster, through TetR-mediated regulation or TeNT biosynthesis itself, modulates global metabolism [32]. Their observation that constitutive expression of TeNT under the ferredoxin (fdx) promoter eliminates native metabolic signatures supports the idea that toxin regulation and redox metabolism are closely interconnected. Despite these observations, the underlying mechanisms remain to be elucidated. Further analyses of the intracellular redox state, enzyme activities, transcriptomics and proteomics should be performed to clarify the mechanisms underlying these observations. During Phase III, the values of r_CO2_ and r_H2_ gradually decreased but remained above zero, indicating that cell lysis did not occur synchronously across the population.

In contrast, strain G761, which lacks the megaplasmid and does not express the TeNT operon, indicated no H_2_ stimulation during Phase II. Its r_H2_ profile remained relatively low and linear, which is consistent with the absence of an enhanced ferredoxin-dependent electron flux. After 80 h, r_CO2_ started to decrease gradually, which coincided with the onset of the biomass plateau (Figure 5B). At this stage, r_H2_ increased slightly, reflecting the uncoupling of metabolic activity from biomass accumulation, possibly due to late hydrogenase induction. A new episode of medium acidification was also observed toward the end of the culture process, indicating a secondary pH transition, potentially resulting from a redox imbalance and promoting H_2_ release.

CH_3_SH production began at the onset of Phase II, with higher production rates in the toxigenic strain, which indicates that methionine metabolism started concomitantly with toxin accumulation. However, because CH_3_SH was also released during Phase II in the plasmid-free strain, a direct causal link between toxin synthesis and methionine metabolism can be ruled out. Nevertheless, a more indirect link between these two mechanisms remains possible.

### 3.5. Metabolites

**Figure 6:**
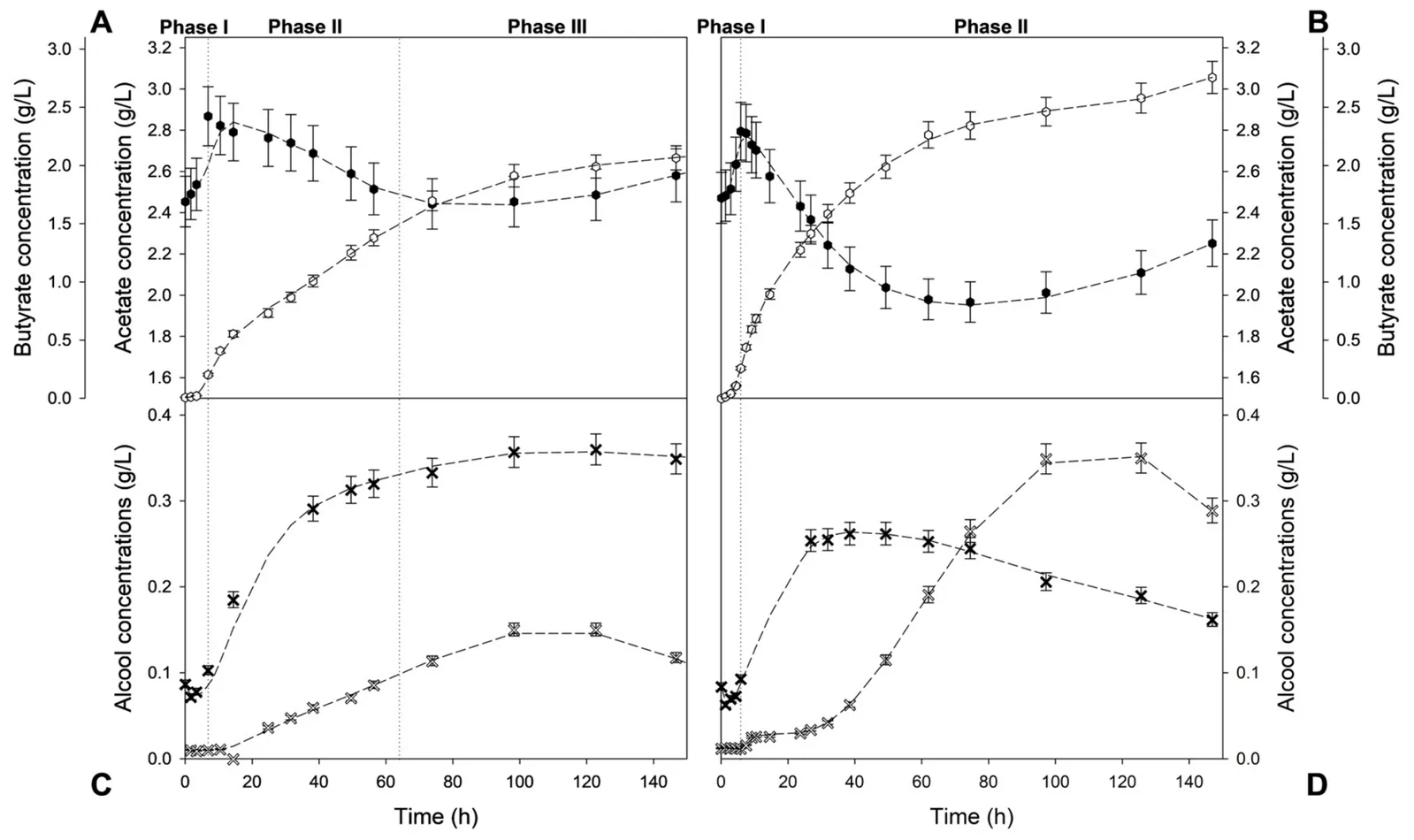
Organic acid and alcohol concentrations from *C. tetani* strain A (**A**,**C**) and *C. tetani* strain G761 (**B**,**D**) in RCM under batch mode culture conditions in a bioreactor. (⬢) Acetate, (⬡) butyrate, (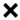) ethanol, (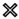) butanol.

The anaerobic fermentation metabolism of *C. tetani* naturally directs the carbon flux toward the production of organic acids, with acetate and butyrate being the most abundant products. Most amino acids, particularly glutamate, and glucose are metabolized into pyruvate. Glucose is subjected to glycolysis to form pyruvate, after which pyruvate is transformed into acetyl-CoA with concomitant CO_2_ release. Lacking the gene of the tricarboxylic acid cycle, but possessing all the enzymes needed for the conversion of acetyl-CoA into butyrate, *C. tetani* obtains energy through ATP formation catalyzed by kinases in the acetate and butyrate pathways [27,29]. Similarly, glutamate, the major amino acid consumed by *C. tetani,* is catabolized into acetate and butyrate, thereby releasing energy in a 2:1 molar ratio, respectively [27,33]. Acetate was already present in the RCM in the form of sodium acetate, which explains the nonzero initial concentration. Both strains yielded very similar production profiles (Figure 6).

Acetate production occurred mainly during Phase I, whereas its concentration decreased upon entry into Phase II in both strains. During this phase, alcohols, primarily ethanol and butanol—were produced. Martinez and Rittenberg (1959) reported that alcohols represent major fermentation products when glucose is used as a substrate [30]. This metabolic behavior can be compared to the alcohologenesis stage, as described for other *Clostridia*. In *C. acetobutylicum*, neutral pH conditions favor the occurrence of acidogenesis (acetate and butyrate production), followed by a switch to alcohologenesis, during which acetate is reduced to butanol and ethanol under the condition of NAD(P)H availability [31].

The transition from Phase I to II has been reported to coincide with the depletion or notable limitation (<10%) of several amino acids [9]. This transition causes a reorganization of the metabolic flux: although acidogenic products continue to be synthesized, a fraction of these organic acids is reduced to alcohols during alcohologenesis, thereby consuming protons and in turn partially alleviating medium acidification [9,31]. In parallel, amino-acid deamination yields the release of ammonium, which contributes to medium alkalization. Together, these processes likely explain the pH increase typically observed at the onset of Phase II.

The alcohol production profiles differed between the two strains. In *C. tetani* strain A, the production levels of ethanol and butanol reached 0.36 and 0.16 g/L, respectively, before the cell lysis was initiated (Figure 6C). In contrast, in *C. tetani* strain G761, butanol predominated, reaching a concentration 0.35 g/L, compared with the maximum value of 0.26 g/L for ethanol prior to the biomass plateau (Figure 6D). In both strains, ethanol accumulated rapidly upon entry into Phase II, followed by a slower increase in the butanol concentration. The contrasting alcohol profiles observed between strains A and G761 may reflect differences in redox management during Phase II, which is associated with enhanced peptide catabolism. Based on the known solventogenic metabolism of *Clostridia* and on the putative metabolic network proposed for *C. tetani*, butanol synthesis is expected to require substantially more reducing equivalents (3 NADH + 1 NADPH per mole) than ethanol synthesis (1 NADH + 1 NAD(P)H) [27,31]. In the toxigenic strain, the energetic and redox burden associated with TeNT biosynthesis may result in the diversion of reducing power away from the butanol pathway, thereby favoring ethanol as a less demanding route for electron dissipation. Conversely, in strain G761, which does not produce TeNT and thus does not incur this additional redox cost, reducing equivalents may be more readily channeled toward the more electron-intensive butanol pathway, thus explaining its predominance.

During Phase III, the onset of cell lysis was accompanied by a metabolic shift characterized by reduced alcohol production, while residual metabolically active cells continued to process substrates, which resulted in a slight increase in the acetate concentration. A similar trend was observed for *C. tetani* strain G761 after approximately 80 h of cultivation. This decrease in alcohol concentrations observed during late culture may have resulted from abiotic processes, such as evaporation under nitrogen flow, adsorption, or chemical oxidation. Although alcohol reconsumption by *Clostridia* has not been reported previously, several metabolic explanations remain plausible. For example, the selective reoxidation of ethanol, which is thermodynamically more favorable and requires fewer cofactors than butanol oxidation, could occur if the redox balance becomes limiting or if hydrogenase activity declines, forcing electrons back toward oxidative pathways. In addition, late H_2_ release may function as a redox valve, thereby counterbalancing reduced alkalinizing deamination activity with renewed acidogenic fermentation, which is consistent with the decrease in pH observed during the final hours of culture. Further investigations are needed to distinguish physical alcohol loss from potential metabolic reutilization mechanisms.

### 3.6. Carbon Mass Balance

**Figure 7:**
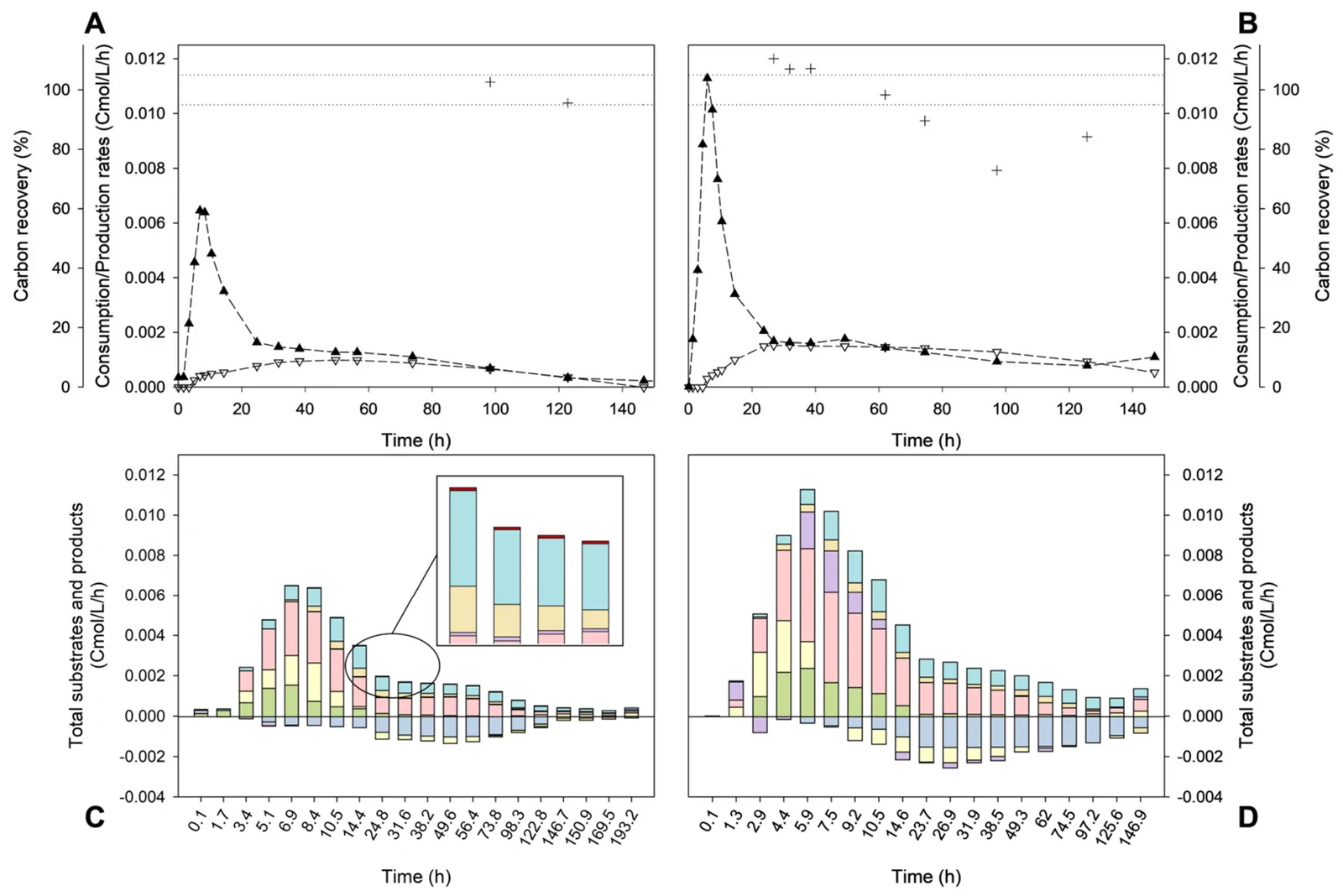
Carbon mass balance and distribution from *C. tetani* strain A (**A**,**C**) and *C. tetani* strain G761 (**B**,**D**) in RCM under batch mode culture conditions in a bioreactor. Most of the carbon recovery not visible is above 110%. (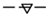) Substrate consumption rate; (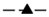) biomass and metabolite production rate; (+) carbon recovery; 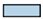 glucose; 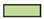 biomass; 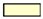 acetate; 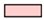 butyrate; 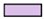 other organic acids; 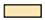 solvents; 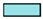 gases; 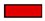 toxin.

The results of carbon recovery analysis clearly indicated that during the first 25 h of cultivation, metabolite formation and biomass production could not be explained by glucose consumption alone. In the *C. tetani* strain A cultures, the carbon balance remained incomplete throughout Phase II, indicating the assimilation of additional carbon sources, such as amino acids and/or peptides (Figure 7A). Protein substrates were preferentially mobilized at the early stages of culture: free amino acids have been reported to be consumed primarily during Phase I, whereas peptide utilization becomes increasingly favored during the Phase I/II transition, which constitutes a shift associated with toxin synthesis [9]. Because peptide utilization is more energy-demanding than free amino acid uptake, this metabolic transition may explain the reduced growth rate observed during Phase II, as reported previously [9]. Histidine, in particular, must be supplied in peptide form (e.g., glycyl-histidine), and toxin production has been shown to correlate with its hydrolysis [34,35,36]. In strain A, the carbon balance became more consistent only upon the onset of cell lysis. However, the direct characterization of amino acid and peptide consumption would be required to fully confirm these interpretations.

In contrast, carbon recovery during Phase II became more consistent in *C. tetani* strain G761, even in the absence of quantitative data on free amino acids or peptides (Figure 7B). At this stage, metabolism was directed mainly toward the conversion of glucose into organic acids and the reduction of acetate into alcohols, reflecting the dominant metabolic activities of this strain during this growth phase.

### 3.7. Cell Distribution

**Figure 8:**
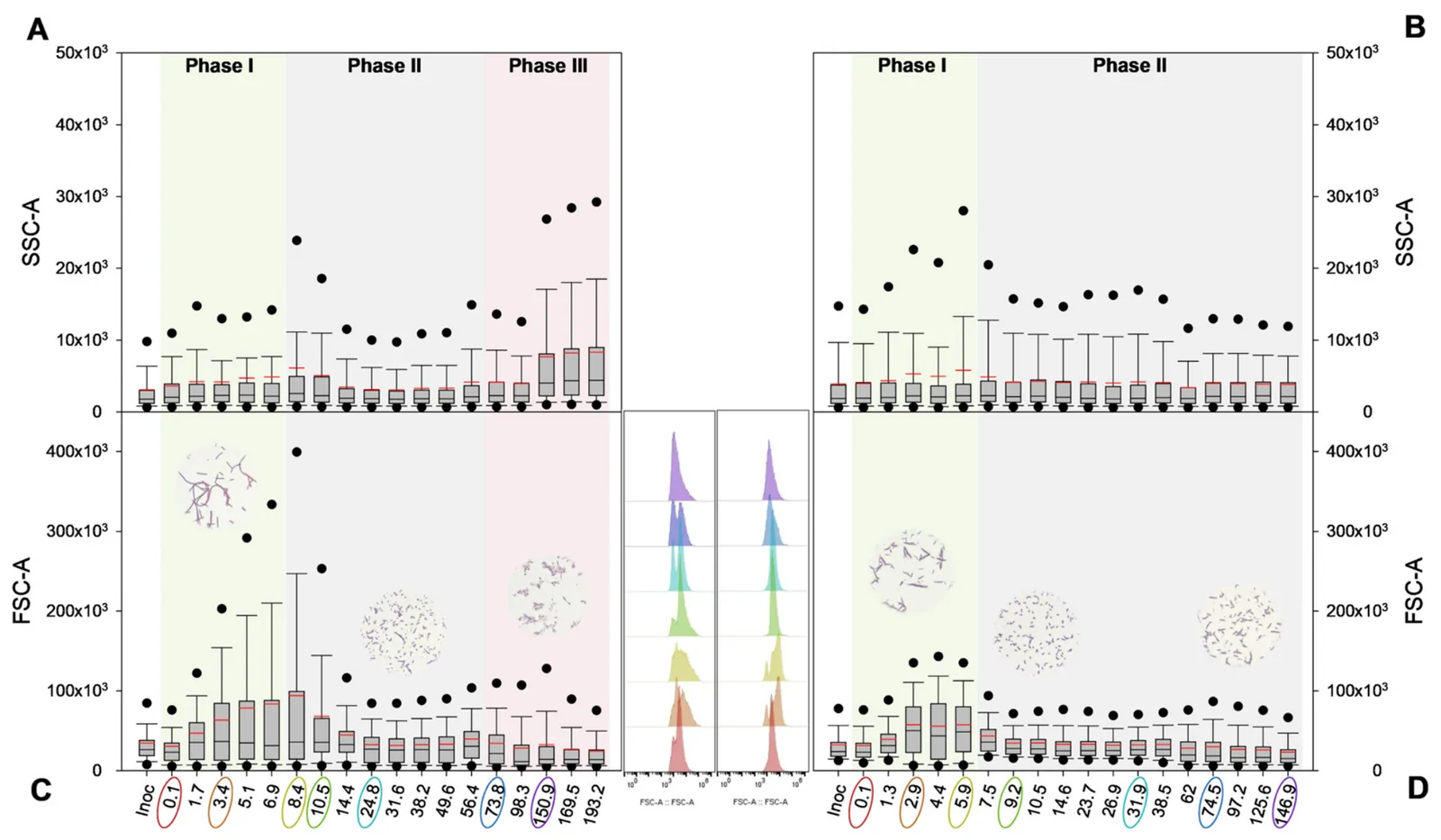
Granulometry (SSC-A signal) and morphology (FSC-A signal) distributions of *C. tetani* strain A (**A**,**C**) and *C. tetani* strain G761 (**B**,**D**) over time during batch culture in RCM. Phases are shown with a microscopic view (GRAM, ×40). The FSC-A signals are also represented as histograms, which show the distribution of the different populations. Red: 0.1 h; orange: 2.9 h; light green: 5.9 h; green: 9.2 h; cyan: 31.9 h; navy blue: 74.5 h; purple: 146.9 h. This graph shows a classical boxplot representation; the boundary of the box closest to zero indicates the 25th percentile, a black line within the box marks the median, a red line within the box marks the mean, and the boundary of the box farthest from zero indicates the 75th percentile. Whiskers above and below the box indicate the 90th and 10th percentiles, respectively. The black dots indicate the 95th and 5th percentiles.

Samples collected from both batch cultures were analyzed through flow cytometry to monitor changes in population distributions at the whole culture level, in terms of both cell morphology and internal complexity, using FSC and SSC signals, respectively (Figure 8).

During Phase I, when the growth rates were high, the cellular morphology was highly heterogeneous, a phenomenon that was less notable in strain G761. Light microscopy revealed the occurrence of filamentous forms, ranging from short bacilli to highly elongated cells. In several filaments, internal septum-like structures were observed, resembling cells in the process of division that had not yet fully separated. This observation is consistent with a septation-associated division process, in which incomplete cell separation can yield a transient filamentous phenotype.

The results of flow cytometry analysis of the FSC-A signal revealed two distinct subpopulations in strain A (Figure 8C, histograms), reflecting notable morphological heterogeneity within the culture. During Phase II, the toxigenic strain exhibited primarily the high-FSC-A subpopulation, consistent with larger or more elongated cells. Such morphologies are typically associated with active growth and may reflect increased metabolic activity. As cultivation progressed toward Phase III, this subpopulation gradually shifted toward lower FSC-A values, indicating a transition to smaller and/or more compact cells. This morphological remodeling coincided with the onset of toxin release and growth deceleration, which suggests that the observed shift reflects a physiological adaptation linked to metabolic transition or stress response rather than direct cell lysis. In contrast, the megaplasmid-free strain G761 exhibited a single, stable FSC-A subpopulation throughout cultivation, indicating the absence of major morphological transitions.

The SSC-A signal, which reflects intracellular complexity, varied moderately during Phase I and at the transition to Phase II in both strains. Thereafter, the SSC-A distribution in strain G761 remained stable throughout cultivation, which is consistent with the absence of significant physiological disruption. In contrast, strain A exhibited a relatively constant SSC-A signal distribution until late Phase III, at which point a marked broadening of the SSC-A signal was observed (Figure 8A). This increase in population heterogeneity coincided with cell lysis in the toxigenic strain, suggesting changes in the intracellular structure that are associated with the lytic process.

Taken together, these observations highlight the major physiological differences between the toxigenic and megaplasmid-free strains. A summary of the principal characteristics identified in this study is provided in Table 1.

## 4. Conclusions

Our results revealed a close coupling between metabolism and physiology in *C. tetani* strain A during tetanus toxin production in RCM. The culture exhibited three distinct growth phases, beginning with a rapid exponential phase (µ = 0.34 ± 0.06 h^−1^). For the first time, the application of flow cytometry revealed pronounced cellular heterogeneity during this phase, with a subpopulation differentiating into elongated filamentous forms. Moreover, microscopic observations further revealed septum-like structures.

During this initial phase, the main fermentation products were acetate and butyrate. Following a metabolic switch demonstrated by a pH shift, growth decelerated markedly (µ = 0.051 ± 0.001 h^−1^). This second phase was accompanied by progressive medium realkalization and the formation of alcohol from acetate, primarily ethanol and butanol. Concomitantly, tetanus toxin also accumulated intracellularly, and a diversification of cellular subpopulations at the morphological level was observed prior to toxin release, which reflects complex structural remodeling of the bacterial population. Notably, membrane integrity remained largely preserved, even when the toxin was identified in the extracellular environment. Although an active or unconventional toxin export mechanism represents one possible explanation, low-level cell lysis or localized cell damage cannot be excluded. Future studies combining transcriptomics and proteomics approaches will be required to identify the molecular mechanisms governing toxin export and the regulatory pathways involved in toxinogenesis.

In contrast, strain G761, which is a nontoxigenic megaplasmid-free variant, exhibited a fundamentally different phenotype. The growth rates were greater during both growth phases, reaching 0.47 ± 0.15 h^−1^ during Phase I and 0.071 ± 0.005 h^−1^ during Phase II. The cell population displayed greater stability in terms of morphology and intracellular complexity. The most striking difference was the complete absence of cell lysis at the end of the culture process, which allowed the cells to reach a relatively high final biomass concentration (0.75 g/L after 100 h) and increased product yields.

The growth deceleration observed a few hours into Phase II in both strains suggests the occurrence of a nutritional limitation. Although RCM is a complex and nutritionally rich medium, it supports the growth of both strains and facilitates toxin production, albeit at moderate levels (<50 Lf/mL). Owing to its ease of preparation, RCM therefore represents a suitable medium for physiological studies of *C. tetani*. More importantly, the comparative experimental framework and flow cytometry monitoring provides a reproducible approach for investigating the physiological determinants of toxinogenesis. This framework should facilitate future studies aimed at elucidating the molecular mechanisms governing toxin production, toxin release, and cell lysis while ultimately supporting the optimization of tetanus toxin production for vaccine manufacturing.

## Figures and Tables

**Table 1:** Comparative overview of the key physiological differences observed between the toxigenic strain A and the megaplasmid-free strain G761 during batch cultivation in RCM medium.

Parameter		Strain A	Strain G761
Megaplasmid		Present	Absent
Tetanus toxin production		45 Lf/mL	-
Biomass (max)		0.45 g/L	0.75 g/L
Growth rate	Phase I	0.34 h^−1^	0.42 h^−1^
	Phase II	0.51 h^−1^ (to 0.005 h^−1^)	0.071 h^−1^ (to 0.005 h^−1^)
Cell lysis (Phase III)		Yes	No
H_2_ formation during Phase II		Increase up to 0.15 mmol/L/h	Low and stable ~0.05 mmol/L/h
Main alcohol		Ethanol	Butanol
Morphology		Heterogeneous + filaments	Homogeneous
Membrane permeability		1.7 → 12% permeable cells (Phase II)	<8% permeable cells at late stationary phase
Carbon balance (Phase II)		125–211%	89–110%

## Data Availability

The original contributions presented in this study are included in the article. Further inquiries can be directed to the corresponding author.
